# Novel Microbial Groups Drive Productivity in an Archean Iron Formation

**DOI:** 10.3389/fmicb.2021.627595

**Published:** 2021-03-30

**Authors:** Cody S. Sheik, Jonathan P. Badalamenti, Jon Telling, David Hsu, Scott C. Alexander, Daniel R. Bond, Jeffrey A. Gralnick, Barbara Sherwood Lollar, Brandy M. Toner

**Affiliations:** ^1^Department of Biology and the Large Lakes Observatory, University of Minnesota Duluth, Duluth, MN, United States; ^2^University of Minnesota Genomics Center, University of Minnesota Twin Cities, Minneapolis, MN, United States; ^3^Biotechnology Institute, University of Minnesota Twin Cities, Saint Paul, MN, United States; ^4^School of Natural and Environmental Sciences, Newcastle University, Newcastle upon Tyne, United Kingdom; ^5^Plant and Microbial Biology, University of Minnesota Twin Cities, Saint Paul, MN, United States; ^6^Department of Earth and Environmental Sciences, University of Minnesota Twin Cities, Minneapolis, MN, United States; ^7^Department of Earth Sciences, University of Toronto, Toronto, ON, Canada; ^8^Department of Soil, Water, and Climate, University of Minnesota Twin Cities, Saint Paul, MN, United States

**Keywords:** geomicrobiology, metagenomics, archean, brines, subsurface, methane

## Abstract

Deep subsurface environments are decoupled from Earth’s surface processes yet diverse, active, and abundant microbial communities thrive in these isolated environments. Microbes inhabiting the deep biosphere face unique challenges such as electron donor/acceptor limitations, pore space/fracture network limitations, and isolation from other microbes within the formation. Of the few systems that have been characterized, it is apparent that nutrient limitations likely facilitate diverse microbe-microbe interactions (i.e., syntrophic, symbiotic, or parasitic) and that these interactions drive biogeochemical cycling of major elements. Here we describe microbial communities living in low temperature, chemically reduced brines at the Soudan Underground Mine State Park, United States. The Soudan Iron mine intersects a massive hematite formation at the southern extent of the Canadian Shield. Fractured rock aquifer brines continuously flow from exploratory boreholes drilled circa 1960 and are enriched in deuterium compared to the global meteoric values, indicating brines have had little contact with surface derived waters, and continually degas low molecular weight hydrocarbons C_1_-C_4_. Microbial enrichments suggest that once brines exit the boreholes, oxidation of the hydrocarbons occur. Amplicon sequencing show these borehole communities are low in diversity and dominated by Firmicute and Proteobacteria phyla. From the metagenome assemblies, we recovered approximately thirty genomes with estimated completion over 50%. Analysis of genome taxonomy generally followed the amplicon data, and highlights that several of the genomes represent novel families and genera. Metabolic reconstruction shows two carbon-fixation pathways were dominant, the Wood-Ljungdahl (acetogenesis) and Calvin-Benson-Bassham (via RuBisCo), indicating that inorganic carbon likely enters into the microbial foodweb with differing carbon fractionation potentials. Interestingly, methanogenesis is likely driven by *Methanolobus* and suggests cycling of methylated compounds and not H_2_/CO_2_ or acetate. Furthermore, the abundance of sulfate in brines suggests cryptic sulfur cycling may occur, as we detect possible sulfate reducing and thiosulfate oxidizing microorganisms. Finally, a majority of the microorganisms identified contain genes that would allow them to participate in several element cycles, highlighting that in these deep isolated systems metabolic flexibility may be an important life history trait.

## Introduction

Like most of Earth’s ecosystems, subsurface environments are vastly under-sampled for microbial life, especially when considering the large diversity of lithologies that occur (Edwards et al., 2012). Earth’s subsurface is estimated to harbor 10^29–30^ microorganisms (Whitman et al., 1998; Kallmeyer et al., 2012; Magnabosco et al., 2018), which in turn sequester 23–135 Pg of Earth’s crustal carbon (Gold, 1992; Whitman et al., 1998; Bar-On et al., 2018; Magnabosco et al., 2018). The large uncertainty associated with biomass and population size is largely driven by estimates of habitable pore size and defining a habitable zone. Difficulties accessing the deep subsurface and problems associated with retrieving pristine samples, have hampered the study of these environments (Colwell and D’Hondt, 2013; Wilkins et al., 2014; Sherwood Lollar et al., 2019). Thus, many first order questions remain regarding the diversity, metabolic activity, connectivity and longevity of these biological systems.

Deep subsurface systems are typically isolated from Earth’s surface and thought to be decoupled from Earth’s surface processes, like photosynthesis (Schrenk et al., 2010). Due to the isolation from the surface, time scales for surface derived water or carbon can range from years to hundreds of millions of years (Lippmann et al., 2003; Lin et al., 2006; Holland et al., 2013). Thus, it stands to reason that deep microbial systems operate on time scales that are counter to what we observe in the lab or at the surface, i.e., growth rates in the lab range from minutes to weeks, while subsurface may see growth on the order of years to decades (Hoehler and Jørgensen, 2013; Xie et al., 2013; Onstott et al., 2014; Trembath-Reichert et al., 2017; Lloyd et al., 2020). As a whole, the subsurface is highly populated with microorganisms. However, there is much uncertainty associated with calculating actual biomass in the subsurface. Integrating these numbers with depth shows that the density of microbes living at depth is quite small (Kallmeyer et al., 2012; Magnabosco et al., 2018). For instance, in a cubic centimeter of rock, microbial communities may consist of a few cells, which may never interact with each other depending on pore size and pore network. Recent work has shown that cell densities in subsurface biofilms can be several orders of magnitude greater than fluids sampled from the formation (Casar et al., 2020). This would suggest that in highly fractured or networked formations cell abundance may be quite large and further highlights that subsurface biomass is likely underestimated and highly variable. However, biomass is still regulated by the presence and flux of electron donors and acceptors to drive microbial metabolism (LaRowe and Amend, 2019). Microorganisms at depth likely employ metabolic strategies that maximize longevity and survivability (Hoehler and Jørgensen, 2013; LaRowe and Amend, 2019), i.e., slow to stagnant growth, operating at or near cellular maintenance energy, or enter into dormancy states (Lennon and Jones, 2011).

For life that thrives in the subsurface, the flux of electron donors and acceptors is key, and dictates the productivity of the system. Early work has shown that sulfate reduction rates peak at shale sandstone interfaces where changes in porosity allow microorganisms to access dissolved carbon leaching from the shale (Fredrickson et al., 1997; Krumholz et al., 1997). More recently, in newly fractured deep shale systems, injection fluids containing microbial osmolytes were shown to stimulate subsurface fermentative organisms that in turn help drive methanogenesis (Borton et al., 2018). In shallow terrestrial settings, push-pull studies show microbial communities rapidly respond to carbon donor and acceptor additions that in turn stimulate the biogeochemical cycling of the community (Istok et al., 2004; Wrighton et al., 2012). In deep terrestrial settings the penetration of paleometeoric water has been shown to be a major factor in supporting higher levels of microbial biomass and activity (Ward et al., 2004; Simkus et al., 2016). Finally, in deep marine sediments, the presence of deeply buried coal deposits correlate with an increase in cell numbers, metabolic activity and community structure when compared to overlying sediments (Inagaki et al., 2015). Together, these studies all highlight that subsurface microbes are metabolically poised to extract energy available in the formation or sediment, however, these systems are also influenced by the presence of surface-derived carbon.

The exploration of subsurface ecosystems, both marine and terrestrial, has shown the subsurface environment holds a wealth of microbial diversity (Sahl et al., 2008; Orcutt et al., 2011; Wrighton et al., 2012; Nyyssönen et al., 2014; Magnabosco et al., 2018; Purkamo et al., 2020). However, we have sampled an extremely small fraction of potentially habitable subsurface systems [as an example see Magnabosco et al. (2018)]. Lithology and the physical conditions (i.e., temperature, salinity, pH, hydrology, pressure, and pore space) play an important role in framing the niche space of microorganisms living in the subsurface. A survey of terrestrial subsurface microbial communities show that microbial cell densities and community structure differ depending on host rock composition (Magnabosco et al., 2018). In hydrothermal and glacial systems the dissolved element composition of the waters are determined by the host rock (German and Von Damm, 2004; Boetius et al., 2015) and thus create the underpinnings for which chemolithoautotrophic metabolisms can be supported. Likewise, in ophiolite deposits the resultant water chemistry constrains the species and metabolic diversity (Brazelton et al., 2012, 2017; Rempfert et al., 2017; Twing et al., 2017). Early work suggested subsurface lithotrophic microbial ecosystems are capable of existing solely on geologically produced hydrogen (Gold, 1992; Stevens and McKinley, 1995; Takai et al., 2004), and indeed there is ample hydrogen in portions of the lithosphere (Sherwood Lollar et al., 2014). However, in many of these systems disentangling purely abiotic versus biological hydrogen production is difficult and suggests that hydrogen is just one facet of the story (Nealson et al., 2005). Nonetheless, as we sample from more subsurface environments, it is becoming apparent that biogeochemical cycling is functionally redundant and is mediated by a diversity of microorganisms (Pedersen, 1997; Osburn et al., 2014; Lau et al., 2016; Magnabosco et al., 2016; Momper et al., 2017).

To date, little work has focused on the microbiology of iron rich geological formations (Pedersen, 1997). However, given the abundance of iron on Earth, understanding how life thrives in environments such as these may give insight into biogeochemical cycles of early earth and potentially subsurface ecosystems on other planets like Mars. Using Soudan Iron Mine as our access to deep subsurface brines entrained in a massive hematite formation, we sought to characterize the microbial communities hosted by the brines. Previous metagenomic work at Soudan Iron Mine has highlighted metabolisms of microorganisms living in mineral crusts and sediments external to but near legacy boreholes used for exploration of hematite (Edwards et al., 2006). However, the locations of these samples were also exposed to oxygen. While this work shows differences in microbial functionality between oxidized and reduced regions, sampling from the borehole brine waters originating from deeper in the formation were not analyzed. Cultivation-based studies from the samples collected within Soudan boreholes have shown microorganisms are capable of iron oxidation (Bonis and Gralnick, 2015) and reduction (Badalamenti et al., 2016), indicating that deeper brines from within the boreholes are metabolically different than those outside of the boreholes. Here we present initial gas composition, water chemistry and microbial analysis of brines, and show that microbial communities are low diversity, phylogenetically novel and metabolically versatile.

## Materials and Methods

### Site Description

**FIGURE 1:**
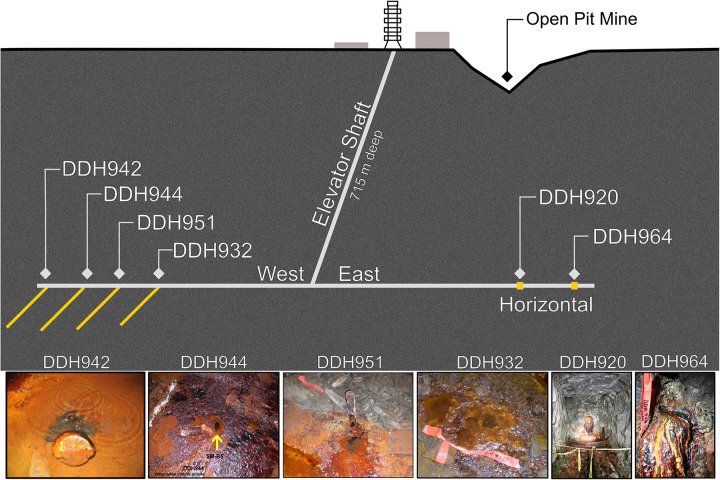
Overview and layout of the legacy boreholes sampled at the Soudan Underground Iron Mine.

Soudan Underground Mine State Park (“Soudan Mine,” 47.8168° N, 92.2489° W) is located in Northern Minnesota, and geologically lies on the southern extent of the Canadian Shield in the Vermilion Greenstone Belt (Peterson and Patelke, 2003). The massive hematite iron formation is Neoarchean (∼2.7 Ga). The geology consists of large broad folds of calcalkaline tholeiitic volcanic strata overlain and locally interdigitated with turbiditic rocks (schists; sericite, chlorite-sericite, and chlorite), locally intruded by gabbroic to felsic porphyry rocks tipped almost on end to an ∼87° dip. Some thin layers and lenses within the schists in Soudan Mine contain graphitic carbon (Cloud et al., 1965). Sampling was performed at the lowest level of the mine (∼715 m below surface) from exploratory boreholes that were drilled while the mine was still operational in the late 1950’s and early 1960’s. Presently, many of the boreholes in the mine have either naturally closed over time from either low brine flow and eventual iron oxide mineral accumulation or were plugged after drilling. Thus, the boreholes from this study were chosen based on flow rates and access. All boreholes are inaccessible to the public and are mostly uncapped (Figure 1). Brine waters are under positive pressure and continuously flow from the boreholes at an average of ∼10–20 ml min^–1^. Boreholes sampled for this study have two spatial orientations, horizontal or angled downward to the north at 50 to 55° (see Table 1 and Figure 1).

**TABLE 1:** Summary of borehole water chemistry from the long-term data.

	DDH-964 (*n* = 3)		DDH-920 (*n* = 7)		DDH-932 (*n* = 7)		DDH-944 (*n* = 7)		DDH-951 (*n* = 7)		DDH-942 (*n* = 8)		Sea water
DDH length (m)	276		76		102		125		144		130		–
orientation	Horizontal		Horizontal		Downward		Downward		Downward		Downward		–

Location	**Eastern**		**Eastern**		**Western**		**Western**		**Western**		**Western**		–
						
	**Mean**	**Deviation**	**Mean**	**Deviation**	**Mean**	**Deviation**	**Mean**	**Deviation**	**Mean**	**Deviation**	**Mean**	**Deviation**	

Temperature (Celsius)	11.4	0.5	10.7	0.5	11.5	0.4	10.9	0.1	10.9	0.2	10.9	0.1	–
pH	6.1	0.7	6.2	0.8	6.0	0.4	5.8	0.5	6.3	0.4	6.3	0.4	–
Conductivity (mS)	78.1	38.0	15.7	6.1	127.0	36.9	113.9	42.3	107.9	30.9	105.0	25.7	–
Oxidation reduction potential (mV)	−62.0	59.4	−66.0	53.5	−150.3	147.2	−210.0	102.5	−178.7	120.6	−467.8	220.3	–
**Cations (ppm)**													
Al	0.1	0.0	0.0	0.0	0.2	0.3	0.1	0.1	0.2	0.3	0.2	0.5	5.3 × 10^–5^
Ba	4.3	0.5	1.6	0.3	9.3	0.5	6.8	0.3	6.6	0.3	6.2	0.3	4.5
Ca	10933.3	378.6	1834.3	12.7	24385.7	898.9	20242.9	594.0	16674.3	545.0	16040.0	879.1	412.8
Fe	33.7	1.6	27.7	7.3	115.7	6.7	95.3	7.3	75.5	24.9	101.7	34.4	Trace
K	177.0	11.4	47.4	1.7	418.6	30.9	354.1	25.4	253.3	34.2	253.6	31.6	399
Li	1.5	0.0	0.3	0.0	2.9	0.3	2.5	0.4	2.0	0.5	2.0	0.5	0.179
Mg	1534.3	40.7	328.3	5.1	2830.0	105.4	2361.4	73.4	2101.4	81.9	1966.1	70.9	1292
Mn	11.2	0.3	1.9	0.0	21.7	0.6	17.6	0.5	15.7	1.0	14.1	0.7	8.4 × 10^–6^
Na	5900.0	132.3	1125.7	20.7	13271.4	457.2	11257.1	496.2	8887.1	288.5	8693.1	233.2	10770
Si	2.8	0.3	3.3	0.5	3.4	1.7	3.0	1.3	3.3	1.5	3.6	2.0	2.8
Sr	287.3	15.0	48.8	1.3	599.1	68.3	506.6	41.3	437.7	17.8	418.9	17.2	7.9
**Anions (ppm)**													
Br	181.3	3.8	33.4	1.1	395.9	7.4	333.4	10.9	271.7	5.5	259.8	7.5	67.11
Cl	34133.3	450.9	6120.0	108.8	73842.9	1550.1	62028.6	747.7	50810.0	1094.2	48591.3	1012.2	19353
F	0.4	0.5	0.2	0.1	0.6	0.4	0.6	0.4	0.6	0.4	0.5	0.3	0.068
NO_2_-N	0.3	0.2	0.0	0.0	0.3	0.2	0.3	0.1	0.3	0.1	0.2	0.1	0.42
NO_3_-N	0.3	0.2	0.0	0.0	0.2	0.1	0.2	0.1	0.4	0.5	0.3	0.3	–
PO_4_-P	0.4	0.5	0.1	0.1	0.6	0.4	0.6	0.4	0.5	0.5	0.9	1.3	0.062
P_total_	0.1	0.0	0.0	0.0	0.2	0.1	0.2	0.1	2.6	3.9	4.0	5.7	0.07
S_2_O_3_	0.8	1.1	0.2	0.1	1.1	0.9	1.1	0.9	1.0	0.9	0.9	0.9	–
SO_4_	69.4	15.0	7.7	2.3	75.5	33.3	78.7	33.9	74.1	33.9	55.4	23.4	2712

*All values are the means taken from several sampling trips spanning several years (December 2004 to January 2013). The number of samplings is represented next to the DDH name. Deviation was calculated as standard the standard deviation. See Supplementary Table 1 for detection limits.*

### Quantification of Brine Chemistry

Brine waters were sampled periodically from December 2004 through January 2013. The archived dataset represents the most continuous record of brine fluid composition to date. Brine temperature, pH, conductance and oxidation-reduction potential were measured using a Thermo Orion A329 multi-meter with the probes: pH 9016BNWP, redox 9678BN, DO/RDO, and temperature/conductance 013005D. For cation and anion quantification, 30 ml of water was collected using a sterile syringe and filtered immediately through a 0.2 μm PES filter (Whatman^TM^). Filtered water was split into two 15 mL plastic tubes for anion analysis and cation analysis. One drop of 6N ACS grade HCl was added to the cation fraction. Major cations (Al, Ba, Ca, Fe, K, Li, Mg, Mn, Na, P, Si, and Sr) were quantified at the University of Minnesota using a Thermo Scientific iCAP 6500 dual view ICP-OES with a Mira Mist Peek nebulizer. Anions (Br, Cl, F, NO_3_^–^, NO_2_^–^, PO_3_^–^, SO_4_^2–^, and S_2_O_3_^2–^) were quantified at the University of Minnesota with a Dionex ICS 2000 – AS19 (4 mm) chromatography column with an ASRS 300 (4 mm) suppressor and a NaOH eluent generator. See Supplementary Table 1 for detection limits of the elemental analysis. Serum bottles (30 ml) were completely filled and capped with butyl rubber stoppers for δ^18^O and δ^2^H analysis. Measurements were made at the Large Lakes Observatory using a Piccaro Model L2130-I, which has precision of 0.03 ‰ for δ^18^O and 0.2 ‰δ^2^H.

### Compositional Gas and Isotopic Analysis

During the December 2006 sampling trip, gases were collected in triplicate by submerging an inverted funnel in the water directly above the sediment where a gas seep of borehole DDH942 forms. At the time of sampling (December 2006), This borehole was chosen due the high output of gasses. Additionally, this borehole is last in the drift and somewhat isolated from the other boreholes (Figure 1). However, there is not direct access to this borehole, as it is semi-plugged (cork) and has a layer of sediment covering it. Gases collecting in the funnel were sampled directly into evacuated 60 ml borosilicate bottles sealed with Bellco thick butyl rubber stoppers via a needle attached to the top of the funnel. Bottles were not pre-flushed prior to evacuation. The butyl rubber stoppers were prepared using the method of Oremland and Des Marais (1983), and the sample bottles pre-fixed with 50 μl saturated HgCl_2_ solution to kill any microbes that may affect gas compositional and isotopic values.

Compositional analyses of gas samples were performed at the Stable Isotope Laboratory at the University of Toronto. A Varian 3400 GC equipped with a flame ionization detector (FID) was used to determine concentrations of CH_4_, C_2_H_6_, C_3_H_8_, i-C_4_H_10_, and n-C_4_H_10_. The hydrocarbons were separated on a J&W Scientific GS-Q column (60 m x 0.32 mm ID) with a helium gas flow and temperature program: initial 32°C, hold 6 min, increase to 220°C at 20°C min^–1^. A Varian 3800 GC equipped with a micro-thermal conductivity detector (uTCD) was used to determine concentrations of H_2_, He, O_2_, and N_2_ (inorganic gases). The inorganic gases were separated using a Varian Molecular Sieve 5A PLOT column (25 m × 0.53 mm ID) with an argon gas flow and temperature program: 35°C for 6 min, increase to 220°C at 20°C min^–1^. Reproducibility for triplicate analyses, from a single bottle, was better than ± 5%.

All isotopic measurements were performed at the University of Toronto. Analyses for δ^13^C were performed by continuous flow compound specific carbon isotope mass spectrometry with a Finnigan MAT 252 mass spectrometer interfaced with a Varian 3400 capillary GC. Hydrocarbons were separated using a 60 m J&W Scientific GS-Q column (60 m × 0.32 mm ID) with the following temperature program: initial 32°C hold for 6 min, increase to 150°C at 5°C intervals, increase to 220°C at 10°C intervals, and hold for 5 min at end. Total error incorporating both accuracy and reproducibility is ±0.5 per mil with respect to triplicate V-PDB standards.

δ^2^H analyses were performed on a continuous flow compound specific hydrogen mass spectrometer with a Finnigan MAT Delta + -XL isotope ratio mass spectrometer interfaced with an HP 6890 GC and a micropyrolysis furnace. Hydrocarbons were separated using a 60 m J&W Scientific GS-Q column (60 m × 0.32 mm ID) with the following temperature program: initial 35°C, increase to 120°C at 5°C increments, increase to 220°C at 10°C increments, and hold for 10 min at end. Total error incorporating both accuracy and reproducibility is ± 5 per mil with respect to triplicate V-SMOW standards.

### Water Filtration, DNA Extraction, and Sequencing

Borehole sampling was performed at two different times in 2012 (amplicons) and in 2014 (metagenome). Six boreholes were sampled during the first sampling in 2012 (DDH-920, DDH-964, DDH-932, DDH-951, DDH-944, and DDH-942) while three boreholes were targeted for metagenomic sequencing in 2014 (DDH-932, DDH-951, and DDH-944). At each sampling time, sterile steel hollow probes were inserted either directly into the boreholes or as deep as possible into the sediments that overly the borehole. For open boreholes, probes were inserted to 30 cm, while sedimented boreholes depth were 2–5 cm. During the 2012 sampling trip waters were filtered through 0.22 μm membrane filters (Millipore) at rates that matched the natural outflow of the boreholes until filters clogged (∼100–500 ml). For metagenomes, waters were filtered using Centramate^TM^ Cassette tangential flow filtration membrane with a 0.1 μm pore size (Pall). For both sampling times DNA was extracted using a standard phenol:chloroform method, resuspended in PCR grade water, and frozen at −20°C until sequencing. All DNA samples were sequenced at the Marine Biological Laboratory, as part of the Census of Deep Life sequencing initiative. Samples from 2012 were sequenced using the Roche GS-FLX Titanium 454 pyrosequencing platform using V6-V4 (518F-1064R) 16S rRNA gene primer set that amplify primarily Bacteria (Thór Marteinsson et al., 2013). DNA for metagenome samples, was quantified using a Picogreen assay (Invitrogen) and then sheared and with a Covaris. Sequencing libraries were generated with the Nugen Ovation Ultralow library protocol. Libraries were pooled at equimolar concentrations and size selected using a Sage PippinPrep 2% cassette. Read insert size was approximately 175 bp to enable read merging. Metagenomes were sequenced using the Illumina HiSeq1000 with 2 × 100 bp paired-end sequencing.

### Amplicon Processing

Amplicon reads were downloaded from the VAMPS web server, which prior to uploading, go through a quality assurance check^1^. To generate operational taxonomic units (OTUs) and taxonomic assignments, reads were processed in Mothur using the 454 protocol (Schloss et al., 2009). Briefly, reads were first screened for homopolymers and reads with ambiguous bases, screened for chimeras with Uchime (Edgar et al., 2011), aligned and position filtered to create reads of similar lengths. OTUs were generated with the Opticlust method using a cutoff of 97% similarity (Westcott and Schloss, 2017), and taxonomy assigned with the Silva 132 database using a Naiive Bayesian method using a cutoff of 70% for reporting a taxonomy (Wang et al., 2007). The potential for DNA extraction kit contamination was assessed using previously described lists (Sheik et al., 2018), however, no obvious contaminants were found remove and no sequences were removed. Microbial diversity and evenness was estimated using the Shannon-Wiener index, Simpson index, and the inverse Simpson index using Mothur. Prior to diversity calculations, samples were rarified uniformly to 15,000 sequences per sample.

### Metagenome Processing, Read Mapping, and Binning

Prior to assembly, Illumina reads were quality-assessed with FastQC^2^, trimmed for quality with Sickle (Joshi and Fass, 2011), trimmed of adapters with Scythe (Buffalo, 2014), and rechecked for quality with FastQC. To cross compare boreholes, reads from each assembly were combined and coassembled with Metaspades v. 3.13 (Nurk et al., 2017) using default settings. Reads were mapped back to assembled scaffolds with BWA-mem (Li and Durbin, 2009). Scaffolds were binned into metagenome assembled genomes (MAGs) with CONCOCT (Alneberg et al., 2014) and MetaBat2 (Kang et al., 2019) using default settings. Genome bins were assessed for quality with Anvi’o (Eren et al., 2015) and CheckM (Parks et al., 2014). Bin refinement and comparison between binning methods was done using Anvi’o. Mean coverage of the genome was calculated with Anvi’o using the reads that were mapped to scaffolds. Because there can be large differences in coverage within a single scaffold, we are using the mean values of all scaffolds calculated for each bin. Genome phylogeny and novelty was assessed with GTDB-TK (Chaumeil et al., 2019), which uses several methods to identify not only the overall phylogeny but can assess how similar the genome is to the reference genomes within the database. Genes within each MAG were annotated with DRAM (Shaffer et al., 2020). DRAM uses KEGG protein Hidden Mark Models (HMMs) (Aramaki et al., 2020) and established thresholds to identify and annotate protein coding genes. Searches of key metabolic genes from DRAM outputs were used to characterize element cycles in MAGs.

### Data Availability

Amplicon data (16S rRNA genes) are available for download from the Visualization and Analysis of Microbial Population Structures webserver (VAMPS)^3^ and the NCBI Sequence Read Archive (SRA); DDH932 (SRX554857), DDH942 (SRX663364), DDH944 (SRX663365), DDH951 (SRX663366), DDH964 (SRX663367), and DDH920 (SRX663368). Metagenome reads are deposited at the SRA project PRJNA340294 and Metagenome Assembled Genomes (MAGs) are available via PRJNA248749.

### Most Probable Number Viable Counts

Samples for Most Probable Number enumeration (MPN) of viable methanogens and viable aerobic alkane oxidizers/H_2_ oxidizers were collected in autoclaved 60 ml borosilicate serum vials, both at DDH942 borehole and at 40 and 90 cm downstream of DDH942 in December 2006. Water samples were collected directly above the gas seep and sealed underwater with a sterile butyl rubber stopper. Sediments were collected using a cut syringe downstream (at 40 and 90 cm) from the main borehole to assess whether sediment populations could utilize the hydrocarbons present from the borehole. Water + sediment samples contained 0.5 ml of sediment added to the water in the bottle via a cut-off syringe prior to sealing. In the case of the methanogens, a small amount of sterile FeS (Brock and O’dea, 1977) was immediately injected into the bottles to ensure anaerobic conditions. Samples were placed on ice immediately and transported to the University of Toronto. All inoculations of the MPN series were performed within 36 h of sampling at the mine.

Methanogen, aerobic short chain alkane oxidizing, and hydrogen oxidizing population sizes were estimated in the water and water + sediment associated with DDH942 (see Figure 1 for picture) using a serial dilution Most Probable Number (MPN) method (Hurley and Roscoe, 1983). The mineral media (final pH = 6) contained (all concentrations are in g L^–1^); NaCl - 1.27, CaCl_2_.6H_2_O - 2.93, MgCl_2_.6H_2_O - 0.05, NH_4_Cl 0.4, KCl 0.1, NaHCO_3_ - 1.72, KH_2_PO_4_ - 0.01, Na_2_SO_4_ - 0.002, with the further addition of vitamins (1 mL L^–1^), trace minerals (10 mL L^–1^), and resazurin (0.1 mL L^–1^), with trace element and vitamin concentrations as per (Edwards et al., 1992). See Supplementary Figure 1 for the MPN experimental design. All MPNs were carried out in 10 ml borosilicate serum vials, sealed with butyl rubber stoppers and capped with aluminum seals. Aerobic MPN media was made and dispensed under air, with additional H_2_ (for hydrogen oxidizing MPNs) with or without a mixture of C_1_-C_4_ alkanes (75% CH_4_, 10% C_2_, 10% C_3_, 2.5% i-C_4_, 2.5% n-C_4_) added to give final headspace pressures of 180 kPa. Controls were done in triplicate and consisted of filtered media with either hydrogen or the alkane mixture. Gas concentrations and mixtures were based on gas composition typically associated with Precambrian Shield settings (Sherwood Lollar et al., 2002, 2006). To quantify methanogen populations, an anaerobic version of the mineral media was made using standard anaerobic techniques with N_2_-purged anoxic water and dispensed in a Coy anaerobic chamber under an anaerobic growth mixture N_2_/CO_2_/H_2_ (80/10/10 v/v). A small concentration (50 μM) of FeS was added to all methanogen MPN vials to ensure anaerobic conditions. MPN vials for autotrophic methanogens were amended with additional H_2_ to give a final headspace composition of H_2_:CO_2_:N_2_ (60:4.5:35.5 v/v) at a pressure of 180 kPa. Two sets of controls were also created in triplicate. MPN vials for heterotrophic methanogens were amended with a combination of 40 mM sodium formate, 10 mM sodium acetate and 10 mM methanol.

Serial dilutions were carried out in triplicate in 7 ml borosilicate vials with thick blue butyl rubber stoppers (Bellco), with an initial inoculum of 0.5 ml into 4.5 ml media, followed by 1:10 serial dilutions to reach a final 10^–5^ dilution. Sterile controls were created in triplicate and consisted of the mineral medium filtered through a 0.2 μm filter. Vials were incubated at 10°C (which is the approximate temperature of Soudan brine fluids (Table 1) for 120 days. In methanogen incubations, headspace gas was analyzed for methane and hydrogen and were considered positive with methane production above negative controls. Similarly, aerobic alkane oxidizers were considered positive by the depletion of alkanes in the headspace. C_1_-C_4_ and H_2_ gases were analyzed by GC (see above methods). Further isotopic characterization of the gas headspace in the aerobic alkane oxidizing enrichments was performed on duplicate positive MPN enrichments’ using methods described above.

## Results and Discussion

### Soudan Brine Characteristics Physical and Geochemical

Soudan Brines are typified by their high salinity, low temperature, slightly acidic pH, and low redox potentials (Table 1). Water temperatures consistently range from ∼10°C to 12°C. The pH of brines is variable depending on the borehole and ranges from 5.2 to 6.03. In downward boreholes that intersect the brines (DDH-932, DDH-942, DDH-944, and DDH-951), major cation concentrations (Na^+^, K^+^, Mg^2+^, and Ca^2+^) are 2–3× greater than contemporary seawater values (Table 1). Conversely, horizontal boreholes (DDH-920 and DDH-964) are less salty and typically have a higher redox potential than downward boreholes. In all boreholes, potential anaerobic electron acceptors, iron and sulfate, are found at similar concentrations, while nitrate and nitrite are undetectable. The isotope composition of hydrogen and oxygen in water taken from the downward boreholes (DDH-932, DDH-942, and DDH-951) show that hydrogen and oxygen isotopes fall slightly above the meteoric water line (Supplementary Figure 2) but are enriched in δ^2^H compared to local meteoric water. Based on their position, these fluids may reflect mixing between brines and local meteoric fluids that have penetrated into the mine environment. Relative to the deep brines of Kidd Creek, which are more elevated over the meteoric line and Soudan brines, represents, for most samples, the absence of significant mixing with local less saline meteoric waters (Li et al., 2016; Sherwood Lollar et al., 2019; Supplementary Figure 2).

Gases collected from the DDH942 borehole were primarily comprised of CH_4_ and N_2_ (Table 2) and are typical of gases identified in the Canadian Shield, Witwatersrand basin in South Africa and the Fennoscandian Shield (Sherwood Lollar et al., 2006). CO_2_ concentrations were below detection limit, as is typical in these highly reducing gases. Higher chain alkanes (C_2_-C_4_) were also detected as well as H_2_ but were minor components (< 2% of the total). For gasses collected from the DDH942 legacy borehole, the ratio of methane to ethane, propane and butane (C_1_:C_2_ + C_3_ + C_4_ or C_1_:C_2_ +) was 62. This value is in a range typical of other Precambrian Shield sites but substantially lower than what is seen for typical microbial produced gases, where low to negligible C_2_ + results in C_1_:C_2_ + ratios of > 1,000 (Sherwood Lollar et al., 2006). Isotopic analysis of the C_1_–C_4_ alkanes (Table 2) show values similar to those identified at Kidd Creek mine but offset in δ^13^C and δ^2^H values for CH_4_ (Sherwood Lollar et al., 2006). The patterns of C_1_:C_2_ + values are suggestive of mixing of a small component of microbially produced methane in a methane pool that is quite similar to the methane and hydrocarbon gases from Kidd Creek that are suggested to be abiogenic in origin. The fact that hydrogen levels for the borehole sampled are below detection limit in Soudan Brines, in contrast to gases in Precambrian Shield settings (Sherwood Lollar et al., 2014), suggests hydrogen scavenging may be occurring. Hydrogen utilizing microorganism are active and/or detected in many subsurface environments (Takai et al., 2004; Nealson et al., 2005; Lau et al., 2016; Momper et al., 2017) and suggests that Soudan microorganisms are poised to use hydrogen as an electron donor. Alternatively, hydrogen scavenging may be due to abiotic oxidation reactions, as more oxidized surface fluids interact with the highly reduced brines during mixing. Regardless, the lack of hydrogen would suggest δ^13^CH_4_ should be more depleted than what is observed at Soudan.

**TABLE 2:** Gas composition, concentrations, and isotopic values for samples taken near borehole 942.

	% volume	δ^13^C	δ^2^H
He	1.37	–	–
H_2_	< 0.04	–	–
O_2_	1.37	–	–
N_2_	27	–	–
CH_4_	70.3	−43.5	−392
C_2_H_6_	1.11	−37.3	−324
C_3_H_8_	0.107	−35.7	−233
i-C_4_H_10_	0.008	−38.7	NM*
n-C_4_H_10_	0.012	−34.2	NM*
Total	101.2	–	–

Hydrogen gas was observed but below detection thresholds and is indicated by the < 0.04%. *NM, not measured.

### Microbial Alkane Oxidation Potential in Brines Downstream of Borehole DDH942

Using brine water only and brine water plus underlying sediment from a transect from borehole DDH 942 (0, 40, and 90 cm downstream), we set up enrichments to detect and quantify methanogens and alkane oxidizing microorganisms. In the methanogenic enrichments (either amended with methanol, acetate or H_2_/CO_2_) no methane was produced after four months of incubation, suggesting methanogenesis is not a dominant metabolism in Soudan brines after exiting the borehole. Past DNA sequencing-based efforts at Soudan from sediments near boreholes also did not detect the presence of methanogens with either 16S rRNA gene clone libraries or from shotgun pyrosequencing reads (Edwards et al., 2006) suggesting that outside the boreholes, methanogens are likely rare community members. No aerobic hydrogen oxidizing bacteria (< 2 cells/ml) were enumerated from any of the samples. In contrast, in the aerobic alkane oxidizing enrichments, we detected the consumption of C_1_- C_4_ alkanes and oxygen after four months of incubation. Alkane oxidizing microorganisms (AOM) were more abundant in the sediment and water enrichments when compared to water only and increased by twofold to threefold with distance from the borehole (Table 3). In headspace gas composition of positive enrichments (*n* = 6) and uninoculated controls (*n* = 4), we detected no decrease in hydrogen or methane. Rather, consumption of O_2_ appears to have been driven primarily from propane, i-butane and n-butane degradation (Table 4). Ethane consumption was variable in the enrichments, as evident by the large standard deviation relative to the controls, but overall was minimal. Additionally, we saw a decrease in the O_2_:CO_2_ in these enrichments suggesting aerobic mineralization from alkane consumption.

**TABLE 3:** Most probable number (MPN) enumeration of aerobic C_1_–C_4_ alkane oxidizing bacteria and hydrogen oxidizing microorganisms.

	MPN’s of AOM (cells/ml)	
	
Distance from borehole DDH 942 (cm)	Water	Water + sediment
0	53–2,900	53–2,900
40	53–2,900	13,000–680,000
90	53–2,900	280–14,000

**TABLE 4:** Headspace gas analysis of aerobic alkane oxidizing MPNs enrichments.

	**Controls (% vol)**	**Positive MPNs (% vol)**	**Relative change (%)**	**Change in δ^13^C in MPNs relative to control**
O_2_	9.70.55	1.992.3	−79.6	–
CH_4_	13.60.34	14.980.66	9.9	−0.10.13
C_2_H_6_	1.770.06	1.810.9	1.8	1.300.07
C_3_H_8_	1.550.05	0.810.4	−47.6	4.350.4
i-C_4_H_10_	0.380.01	0.310.04	−15.6	0.730.1
n-C_4_H_10_	0.270.01	0.090.08	−66.4	2.60.5
H_2_	21.770.9	22.70.9	4.3	–
CO_2_*	4.96	7.2	46.6	–

*The number of control bottles measured (*n* = 4) and the number of positive MPN bottles measured (*n* = 6). For δ^13^C measurements (One control bottle and two positive MPN bottles), values represent the change in δ^13^C between MPNs and uninoculated control bottles. Estimated by comparing the size fo the CO_2_ peak on mass spectrometer trace to that of the CH_4_ peak.*

To identify whether the δ^13^C of the alkanes were altered in the enrichments, we selected two positive and one control bottle and analyzed the isotopic composition of the headspace gases. For methane and i-butane there was no change in δ^13^C relative to the controls (Table 4), but δ^13^C values for ethane, propane, and i-butane and n-butane became more positive. Together, this may indicate microbial consumption of these higher molecular weight alkanes with the classic ^13^C-enrichment in the residual pool. As for methane, despite it being highly abundant in bulk gas from the boreholes, it is unclear why no consumption was observed. Nonetheless, it appears that once the short chain alkanes exit the boreholes, microorganisms at the sediment water interface are primed to consume them.

### Down Borehole Microbial Community Diversity

Microbial communities in the horizontal boreholes were less diverse and more evenly distributed (Shannon and Simpson index, respectively) than the downward boreholes, where waters are more saline (Table 5). From our sampling it is difficult to interpret why the orientation of the boreholes would influence the diversity. One possibility is that the drill line through the formation does not intersect veins of rock containing chemosynthetic energy sources that would drive microbial productivity. A second possibility is that some highly abundant organisms prefer lower salinity environments, which allow them to thrive in these boreholes. In the eastern drift brines, waters are much less salty than the western drift, as it intersects a different formation (Table 1). The differences between the two formations is likely driven by the structural orientation of the formation that allows meteoric waters to mix more rapidly with deeper brines. Finally, it should be noted that with few highly abundant OTUs the ability to detect of rare OTUs decreases. Thus, the decrease total diversity could be due to the sequencing read depth per sample (rarified to 15,000 seqs per sample) and not necessarily that these boreholes harbor less diversity.

**TABLE 5:** Diversity of borehole microbial communities. Boreholes are ordered (Left to right) to correspond with table one and signify whether the boreholes are horizontal (DDH-920 and DDH-964) or downward (DDH-932, DDH-944, DDH-951, and DDH-942).

	**DDH-920**	**DDH-964**	**DDH-932**	**DDH-944**	**DDH-951**	**DDH-942**
No. reads	23582	22667	22841	22939	19680	18659
Rarified depth	15000	15000	15000	15000	15000	15000
No. OTUs	4044	4134	4372	4641	5790	3950
Shannon entropy	4.63	4.63	4.96	5.15	5.67	5.01
Simpson	0.13	0.13	0.07	0.06	0.05	0.05
Inverse simpson	7.73	7.5	14.88	15.75	20.38	20

### Borehole Bacterial Community Structure

**FIGURE 2:**
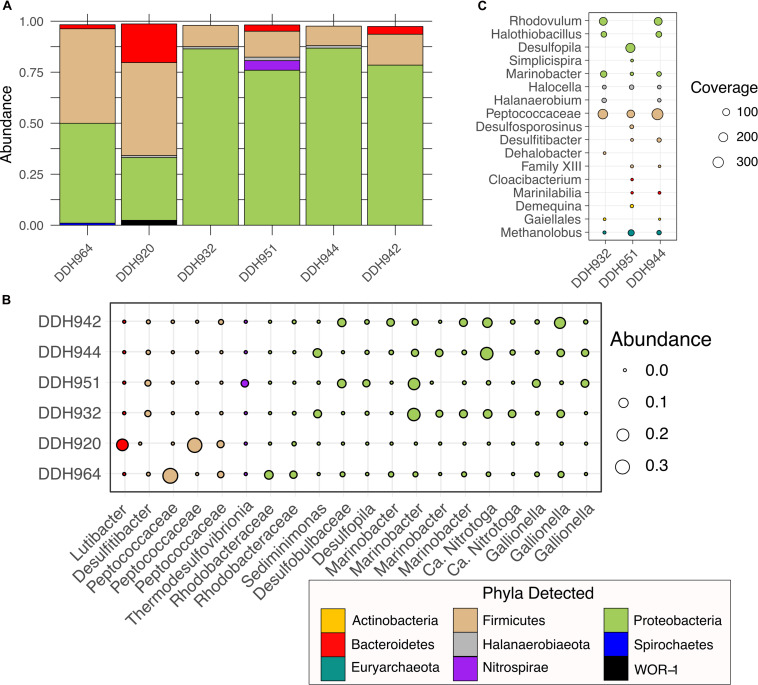
Phylum and OTU_0.03_ level diversity in borehole microbial communities. **(A)** Abundance of phyla recovered from pyroTag sequencing, **(B)** Individual OTU_0.03_ abundance, and **(C)** Total coverage by phyla for 16S rRNA genes assembled from the shotgun metagenome raw reads.

At the phylum-level, Soudan borehole communities are generally represented by the same three phyla, Proteobacteria, Firmicutes, and Bacteroidetes (Figure 2A). Magnabosco et al. (2018) highlight that Firmicutes and Proteobacteria are the most commonly encountered bacterial phyla in the subsurface. Horizontal boreholes (DDH964 and DDH920) contrasted with downward boreholes (DDH 932, 942, 944, and 951) in the proportion of Proteobacteria and Firmicutes, whereby the downward boreholes had higher proportions of Proteobacteria and horizontal boreholes had higher Firmicutes (Figure 2A). Bacteroidetes were present in three of the six boreholes and in DDH920 were nearly as abundant as the Proteobacteria. Less abundant phyla identified were Nitrospirae, Halanaerobiaeota and Spirochaetes. Downward boreholes (DDH-932, DDH-951, DDH-944, and DDH-942) were heavily dominated by Proteobacteria (Figure 2A) and taxonomically spread across Alpha-, Beta-, Delta-, and Gammaproteobacteria. No Zetaproteobacteria were detected despite the salinity of the environment. *Marinobacter* were abundant in the downward boreholes but were most dominant in DDH-951 and DDH-932 (Figure 2B). We recovered several *Marinobacter* OTUs, of which the most abundant OTU identified was 98.9% similar to *M. subterrani.* Previously, *Marinobacter subterrani* was isolated from waters near borehole DDH942 and was shown to be capable of iron (II) oxidation but had no carbon fixation potential, suggesting a counterintuitive form of iron metabolism (Bonis and Gralnick, 2015). Further, *Marinobacter* have been identified in deep shale ecosystems and is well adapted to high-salt ecosystems, suggesting this metabolically diverse genus may be important in the subsurface (Daly et al., 2016). Boreholes DDH-942 and DDH-944 were also abundant in *Nitrotoga* and *Gallionella*. *Nitrotoga* have been shown to be nitrite oxidizing organisms (Boddicker and Mosier, 2018; Kitzinger et al., 2018) while *Gallionella* are associated with iron oxidation under micro-aerophilic conditions (Hanert, 2006). The presence of both these organisms would suggest that samples may have been taken at very shallow depths, where mineral crusts or sediments impede access to deeper regions of the borehole and oxygen was able to penetrate. In the horizontal boreholes, Firmicute abundance was due primarily to Peptococcaceae OTUs (Figure 2B). Interestingly, these OTUs were differentially abundant between DDH-964 and DDH-920, and were rare or absent community members in the downward boreholes. Additionally, two Rhodobacteraceae OTUs (Alphaproteobacteria) were also abundant in DDH-920 and less in DDH-964 horizontal boreholes.

In the metagenome, assembled 16S rRNA gene (Figure 2C) diversity was much less than observed with the PyroTags but showed similarity with the dominant OTUs recovered (Figure 2B). Peptococcacaea sequences were present and dominant in all three boreholes, while *Marinobacter*, *Halocella*, and *Methanolobus* were present in all three samples but not as abundant. The presence of *Methanolobus*, suggests that methylated compounds may be one of the sources of methane, as this genus of methanogen has not been shown to use either H_2_/CO_2_ or acetate (Evans et al., 2019). In shale systems, similar methyl-utilizing methanogens are present and consume methylamines produced by Firmicutes (Daly et al., 2016). In the PyroTags and the metagenomes we detected organisms belonging to the Halanaerobiales, suggesting that similar processes may occur in Soudan brines as in deep shales. However, neither the genomes or the PyroTags were similar to the *Frackibacter* (Daly et al., 2016). Daly et al. (2016) highlight that not all *Halanaerobium* genomes contain the ability to create methylamines from glycine-betaine, thus further examination of these genomes are needed.

### Characterization of Metagenome Assembled Genomes

Through the Census of Deep Life three metagenomes were generated from three separate boreholes, DDH-932, DDH-944, and DDH-951. Using a combined assembly, a total of forty Metagenome Assembled Genomes (MAGs) were recovered. Using CheckM (Parks et al., 2014), we found that twenty-three had estimated completion over 50% and contamination near or below 10% (Supplementary Table 2). Many of the MAGs with very low completion under 10% were also recovered and are likely orphan bins of more complete bins or represent strains of more complete MAGs. Several of the MAGs, despite low contamination, were also high in strain heterogeneity (see Supplementary Table 2, Soudan-18, Soudan-11, Soudan-14, and Soudan-3). This would suggest that some species may exhibit high strain-level diversity between the boreholes, and because we used a co-assembly the MAGs strain-level diversity may be more pronounced (Chen et al., 2020).

**FIGURE 3:**
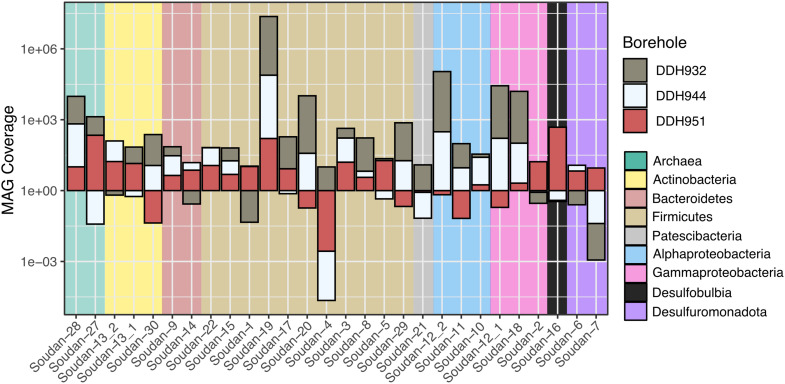
Taxonomy and coverage of Metagenome Assembled Genomes (MAGs) recovered from Soudan metagenomes. Bars below one represents rare to nearly absent genotypes.

Metagenome assembled genomes taxonomy patterns were similar to the pyroTag datasets and show that MAG abundance is variable across the boreholes (Figure 3). The most recovered Phylum were the Firmicutes (11 MAGs) followed by Proteobacteria, Alpha (3 MAGs) and Gamma (3 MAGs). Additionally, two near-complete *Methanolobus* MAGs (Figure 3 and Supplementary Table 2) were identified, confirming their presence from the assembled 16S rRNA gene libraries. One MAG, Soudan-6, is likely from laboratory contamination, see below in the section “Iron.”

Interestingly, many of the genomes were determined taxonomically novel by GTDB at either the species, genus or family (Supplementary Table 2), and one of the Firmicutes, Soudan-17, genome is novel at the Order level. Average Nucleotide Identity (ANI) and Amino Acid Identity (AAI) of the MAGs that share a similar lineage, such as Soudan-3, Soudan-5, and Soudan-8, indicate these genomes likely represent individual species as the ANI values were all below the proposed 95% cutoff for species (Jain et al., 2018). The novelty of many of these genomes likely stems from a general under sampling in deep terrestrial systems and a dearth of genomes in databases like GTDB.

We observed a high degree of variability in genome coverage between the boreholes (Figure 3). We are using genome coverage as a proxy for abundance and is the mean number of mapped reads that overlap at a given nucleotide position on the assembled contig. We observed similar trends in the amplicon datasets as well, when looking at individual OTUs (Figure 2B). This variability may be attributed in part to the orientation of the formation. The formation of Soudan is nearly vertical [∼87° (Peterson and Patelke, 2003)] and so the boreholes, despite their relative closeness, likely do not intersect the same mineral veins or waters. Thus, the variability we observe in genome abundance is likely driven in part by the abundance of electron donors/acceptors available along the borehole. Additionally, abundances may be driven by species-species interactions (Lau et al., 2016) or viral predation (Daly et al., 2019). Nonetheless, a majority of the MAG were detected in all three boreholes with Soudan-19 (Firmicute) and Soudan-28 (Methanogen) being the most evenly distributed amongst the three boreholes.

### Carbon Fixation and Fermentation in Soudan MAGs

CO_2_ fixation is a keystone process in the subsurface environment and unless there are other carbon sources preserved in the rock formation, like kerogen or graphite, will serve as the baseline δ^13^C fractionation for subsequent trophic interactions. We found two primary carbon fixation pathways were present in the Soudan MAGs, Wood-Ljungdahl (Acetogenesis or WL) and the Calvin-Benson-Bassham (CBB) pathways (Supplementary Figure 3). A total of nine MAGs contained the WL pathway, primarily the Firmicutes but also one Actinobacteria and the Desulfobacterota, while five MAGs contained the CBB, two *Methanolobus*, one Actinobacteria, Alphaproteobacteria and Gammaproteobacteria (Supplementary Figure 3). We should note that the two *Methanolobus* MAGs contain both the WL and a portion of the CBB pathway. In the case of the CBB pathway, it has been posited that the missing phosphoribulokinase (PRK) gene prevents Archaea from using the *bona fide* Ribulose 1,5 Bisphosphate Carboxylase/Oxidase (RuBisCO) in the canonical carbon fixation pathway [see references within (Berg et al., 2010)]. However, evidence of PRK homologs in some Archaea suggests the RuBisCO may be used for AMP recycling while still fixing CO_2_ (Sato et al., 2007) or as observed in some methanogenic archaea to function canonically (Kono et al., 2017). The RuBisCO genes from the Actinobacteria, Alphaproteobacteria, and Gammaproteobacteria MAGs are *bona fide* and phylogenetically fall into form I and II. None of the key reverse tricarboxylic-acid cycle (rTCA), 3-Hydroxypropionoate/4-hydroxybutyrate or the 3-Hydroxypropionate cycles genes were detected in our MAGs. This would suggest that they are not used by microorganisms in our system or that our sampling scheme and depth of sequencing (number of reads generated per sample) did not adequately access less abundant organisms down borehole. The rTCA cycle has been detected in other deep subsurface systems (Lau et al., 2016) and given the energy efficiency of the rTCA cycle (Mall et al., 2018) it is surprising we do not observe it at Soudan. We cannot rule out the usage of the normal TCA cycle operating in reverse (Mall et al., 2018), as it cannot be distinguished bioinformatically and is solely detected using culturing-based approaches.

### Methanogenesis

As mentioned previously, two, near-complete *Methanolobus* MAGs were recovered, and are 71% similar by Average Amino Acid Identity (AAI). These methanogens are typified by their inability to use acetate or H_2_/CO_2_ and prefer C_1_ and methylated substrates (Thauer et al., 2008; Evans et al., 2019). Searches of the unbinned contigs for methyl coenzyme M reductase (*mcrA*) and 16S rRNA genes recovered no genes associated with either acetoclastic or hydrogenotrophic (H_2_/CO_2_) methanogenesis. Again, attempts to enrich for H_2_/CO_2_ methanogens at Soudan were unsuccessful. While we cannot rule out alternative styles of methanogenesis due to depth of sequencing, low concentrations of H_2_ in borehole gases (Table 2) would suggest that other terminal electron accepting processes, such as Fe (III), Mn (IV), and SO_4_^2–^ reduction (Lovley and Goodwin, 1988) or abiotic processes could be removing hydrogen. It has been suggested that salinity of subsurface fluids may promote methylotrophic methanogenesis, as it is more thermodynamically favorable per mol substrate than acetoclastic or hydrogenotrophic methanogenesis (Waldron et al., 2007; Oren, 2011).

**FIGURE 4:**
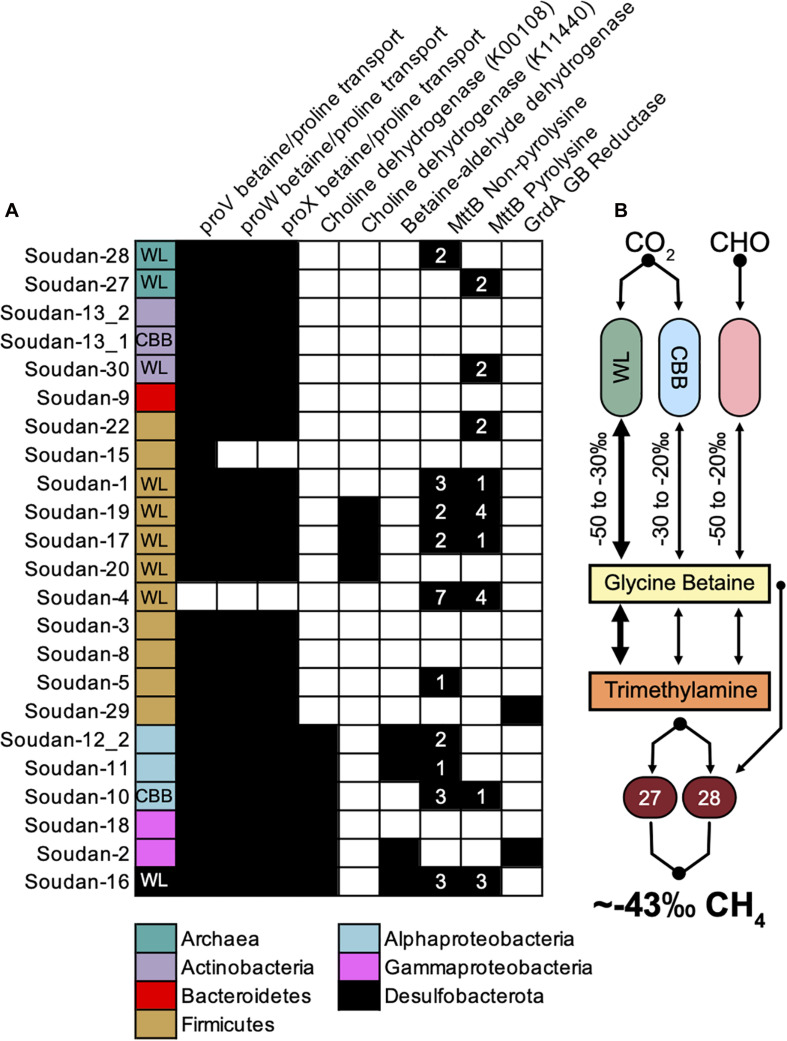
Potential routes for Trimethylamine (TMA) production and isotopic fractionation of methane. **(A)** MAGs containing genes for glycine betaine uptake, glycine betaine production, and glycine betaine conversion to trimethylamine (left to right). Colored boxes represent phyla association and WL and CBB inset indicate the presence of the Wood-Ljungdahl or Calvin-Benson-Bassham cycle. **(B)** Theoretical flow of carbon and the potential isotopic fractionations that would render the methane signature of Soudan.

Recently in hydraulically fractured, deep shale environments (Daly et al., 2016), it has been shown that glycine-betaine (GB), which is a common microbially synthesized osmolyte in high salinity environments (Sleator and Hill, 2002), is produced and fermented to trimethylamine, which can then fuel methanogenesis. A majority of Soudan MAGs (22 total) contained glycine betaine/proline ABC transport system (*proVWX*) including the *Methanolobus* (Figure 4A). We identified nine MAGs that contained genes for producing GB (Figure 4, Choline dehydrogenase and Betaine-aldehyde dehydrogenase) and three MAGs that potentially use the glycine/sarcosine methyltransferase (not shown in Figure 4). We identified only one MAG (Bin 10) that has the ability to produce GB and uses the CBB cycle for carbon fixation. In contrast, there are four Firmicute GB producers that use the Wood-Ljungdahl pathway. The *Methanolobus* MAGs contained no genes for creating glycine-betaine suggesting it relies solely on the scavenging from the environment. Together, this indicates that microbes living at Soudan are poised to scavenge exogenous GB released into the environment through cell death or viral lysis (Daly et al., 2016) but relatively few organisms are capable of producing GB.

Once produced or assimilated by the microbe, glycine betaine can be converted to trimethylamine from two mechanisms, a glycine/sarcosine/betaine reductase via a Stickland fermentation reaction (Stickland, 1934) or using a non-pyrolysine containing glycine betaine methyltransferase homolog (MttB). Recent work has shown that this MttB homolog is able to convert GB to dimethylglycine and subsequently trimethylamine via the Wood-Ljungdahl pathway (Ticak et al., 2014; Daly et al., 2016). We identified two bins that have the GB reductase (Soudan-2 and Soudan-29). Phylogenetic analysis of MttB annotated proteins revealed ten MAGs that contain the non-pyrolysine MttB homolog, which include four Firmicutes and one *Methanolobus* (Figure 4A). Several MAGs encoded multiple copies of both versions of the putative MttB protein, suggesting GB may be converted to trimethylamine and demethylated to dimethylamine. Interestingly, one *Methanolobus* MAG (Soudan-28), contained the non-pyrolysine *mttB gene* homolog while the other encoded the pyrolysine *mttB gene.* This would suggest that Soudan-28 may be able to directly produce methane from GB, while Soudan-27 may require exogenously produced trimethylamine. This, in part, may account for the abundance and distribution differences of the *Methanolobus* observed between the boreholes (Figure 3), as Soudan-27 may rely on syntrophic interactions (Lau et al., 2016) or specific species interactions for access to trimethylamine.

At Soudan, microbial methane is most likely being generated using methylated compounds and potentially through glycine betaine intermediates. In our system we only detected two potential carbon fixation pathways, the Calvin-Benson-Bassham (CBB) and the Wood-Ljungdahl (WL). We know from other studies that typical δ^13^C values for the WL pathway range from −50 to −30‰ while CBB fixation is more enriched in δ^13^C in the −30 to −20‰ (Berg et al., 2010). Using these ranges the bulk isotopic composition of GB could range from −50 to −20‰ for newly fixed and fresh GB from primary producers. However, it is uncertain what the δ^13^C fractionation from heterotrophic GB production would be, as carbon could be coming from many carbon pools each with a different δ^13^C source signature. We observed only one MAG (Soudan-10) that uses the CBB pathway and contains genes for producing GB. Conversely, we found five MAGs with high genome coverage (Figure 3) that utilize the WL pathway and contain GB production genes. Thus, we propose that Wood-Ljungdahl (WL) pathway is likely the primary carbon fixation route for generating GB that fuels methanogenesis. As noted, the isotopic compositions and C_1_:C_2_ + ratios at Soudan are similar to end-member gases described at the geologically similar setting of Kidd Creek but with mixing of microbially produced methane. A subsequent and more detailed study of methane using clumped isotopologue methods could help detangle the sources and cycling of methane at Soudan (Young et al., 2017). Finally, the presence of GB fermentation processes in deep shale systems (Daly et al., 2016; Borton et al., 2018), suggests that in saline subsurface environments GB cycling is an important metabolism that should not be overlooked.

### Sulfur Cycling in Soudan Brines

**FIGURE 5:**
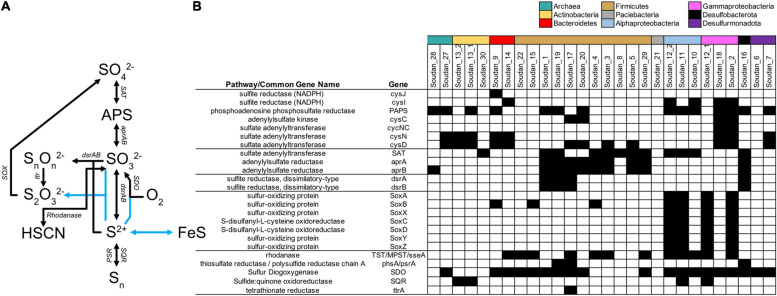
Sulfur cycling genes detected in metagenome assembled genomes (MAGs). Sulfur biogeochemical cycling pathways **(A)** interpreted from genes detected in Soudan MAGs **(B)**. Blue lines represent abiotic reactions.

Sulfur cycling is a primary mode of metabolism in the subsurface (Baker et al., 2003, 2015; Chivian et al., 2008; Anantharaman et al., 2016, 2018; Lau et al., 2016; Jungbluth et al., 2017; Momper et al., 2017), and at Soudan sulfur reduction was anticipated, as there are visible sulfide deposits at the orifices of several boreholes, a presence of sulfide odor at some boreholes and sulfate present in the outflow waters. At Soudan, a previous metagenome survey (Edwards et al., 2006) and an electrode enrichment MAG (Badalamenti et al., 2016) only detected portions of the assimilatory sulfate reduction pathway. Here we identified multiple MAGs with sulfur assimilation pathways (PAPS, *cysCDNIJ*, Figure 5) and several MAGs with a sulfate adenylyltransferase (SAT) and adenylylsulfate reductase (aprAB) which can be used in assimilation, oxidation or dissimilatory reduction. We also identified four MAGs, Soudan-16 (*Desulfobulbaceae*), Soudan-17 (novel *Moorellia*), Soudan-19 (*Desulfotomaculum*), and Soudan-1 (*Desulfosporosinus*) with sulfite reducing capabilities (*dsrAB*). Of these four, only Soudan-17 did not contain genes encoding the SAT. However, Soudan-17 also contained genes for the initial portion of the assimilatory sulfate reduction to sulfite (*cysND*), which may be able to take the place of the SAT. All four MAGs encoded the dissimilatory sulfite reductase *dsrABCDJMOP* and Soudan-17 and Soudan-1 contained the additional *dsr*T. To date, several sulfur reducing families are present within the Firmicutes and the identification of the novel Moorellia MAG as a potential sulfur reducing organism adds to this phylum’s broad metabolic capability. The phylogenetic placement of MAG Soudan-16 shows that it belongs to a novel genus of *Desulfobulbales* (Supplementary Table 2). Several members of the *Desulfobulbaceae*, despite containing only the reduction style *dsrAB*, oxidize sulfide by reversing the pathway (Dannenberg et al., 1992; Kuever, 2014; Kjeldsen et al., 2019) or by disproportionating S^0^ to sulfide and ultimately sulfate (Müller et al., 2020). These organisms are also able to couple the oxidation of sulfur to other electron donors, like nitrate and oxygen. Cable bacteria, which belong to the *Desulfobulbaceae*, also potentially utilize conductive filaments to transport electrons over large distances to couple sulfur oxidation to oxygen reduction (Müller et al., 2020). Interestingly, Soudan-16 also contains nitrate reduction pathway genes *nrfA*, suggesting, that it may be capable of coupling sulfur oxidation to nitrate reduction, as is seen for *Desulfurivibrio alkaliphilus* (Thorup et al., 2017). However, isolation and further experiments are necessary for elucidating whether this organism is capable of nitrate coupled sulfur oxidation.

Potential routes for sulfur oxidation from sulfides are limited at Soudan. We identified no MAGs containing the reverse style of the *dsrAB*, which is an indicator of sulfur oxidizing microorganisms (Loy et al., 2009, 2012). We did, however, detect sulfide:quinone oxidoreductase (SQR) that can create or utilize polysulfides (S_n_^–^) from sulfide in MAGs, Soudan-11, Soudan-12_1, Soudan-13_1, and Soudan-13_2 (Figure 4). Additionally, three MAGs contained a polysulfide reductase. We detected no sulfur oxygenase reductase (SOR) that are capable of disproportionating S^0^ to sulfide, sulfite and thiosulfate. Four MAGs (Soudan-2, Soudan-12_1, Soudan-11, and Soudan-12_2) contain the full SOX thiosulfate oxidizing pathway (Friedrich et al., 2005). Phylogenetically, the MAGs are associated with Gammparoteobacteria (*Marinobacter* and *Halothiobacillus*) and the Alphaproteobacteria (*Confluentimicrobium)*. Previous work from subsurface environments has shown that some *Marinobacter* (Choi et al., 2009; Rani et al., 2017) and the *Halothiobacillus* (Whaley-Martin et al., 2019) are capable of sulfur oxidation, while this is the first instance of a *Confluentimicrobium* sp. putatively capable of sulfur oxidation. Past subsurface metagenomic and metatranscriptomic surveys have shown SOX genes to be present (Anantharaman et al., 2016) and transcriptionally active (Lau et al., 2016). At Soudan, we have detected thiosulfate but at very low concentrations (Table 1), indicating there must be biological or abiotic oxidation routes for thiosulfate formation and further suggest that thiosulfate may be an important oxidized sulfur species in the subsurface, as it can be oxidized to sulfate, disproportionated or directly reduced by microorganisms. Finally, nineteen MAGs contained a sulfur dioxygenase (SDO) that when in the presence of S-sulfanylglutathione (which forms spontaneously from glutathione disulfide and polysulfides) and oxygen can create sulfite (Liu et al., 2014). The prevalence of this enzyme in our MAGs, suggest the enzyme may be used to detoxify O_2_ that is generated through radiolytic processes (Li et al., 2016), rather than detoxifying sulfide as is generally assumed (Liu et al., 2014).

### Nitrogen Cycling in Soudan Brines

**FIGURE 6:**
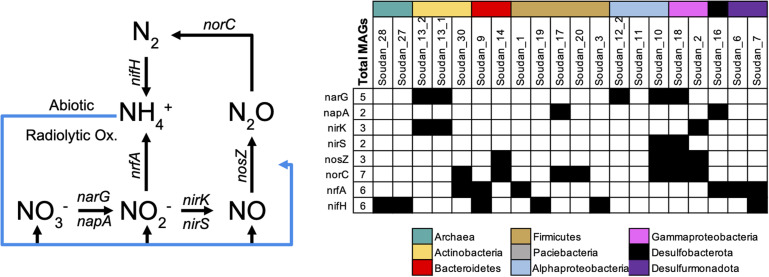
Nitrogen cycling genes detected in metagenome assembled genomes (MAGs). Nitrogen biogeochemical cycling pathways (left) interpreted from genes detected in Soudan MAGs (Right). Blue lines represent abiotic reactions.

Previous subsurface work has shown that nitrogen cycling is important and active in shallow and deep subsurface environments (Chivian et al., 2008; Swanner and Templeton, 2011; Silver et al., 2012; Anantharaman et al., 2016; Lau et al., 2016; Momper et al., 2017). At Soudan, oxidized nitrogen species, nitrate and nitrite, have routinely been measured but are typically undetectable in brines (Table 1). Likewise, ammonia has not routinely been measured from all boreholes at Soudan, but it has been detected at or below 1ppm. In the metagenomes we only detected MAGs with the ability to fix or reduce nitrogen (Figure 6). No genes or organisms capable of nitrification (NH_4_^+^ → NO_3_^–^, or NH_4_^+^ → NO_2_^–^ → NO_3_^–^) or anaerobic ammonia oxidation (anammox) were detected in either the 16S rRNA amplicons or assembled MAGs. The absence of oxidizing organisms is likely due to the reducing potential of the brines, which can scavenge and maintain oxygen concentrations below physiologically relevant concentrations, thereby preventing organisms from using nitrifying pathways that require oxygen. Without microbial oxidation pathways being present, it would suggest that abiotic oxidation processes, such as Fe(III) oxidation of ammonia (Doane, 2017) or radiolytic oxidation of ammonia (Silver et al., 2012), are maintaining an oxidized pool of nitrogen. The presence of nitrogen reduction genes (Figure 6) in many of the MAGs suggests nitrate and nitrite concentrations could be much higher in our brines and that reduction process are driving the concentrations to near detection limits. Here we observed dissimilatory reduction pathways that can generate either N_2_ or NH_4_^+^ (Figure 6). Two MAGs (Soudan- 10 and Soudan-18) contained genes encoding a full nitrate dissimilatory pathway (i.e., *Nar/Nap, nirK/S, NosZ*, and *Nor*), and a third (Soudan-2) was missing only a nitrate reductase, suggesting it may either prefer nitrite or the nitrate reductase is present but was not binned. As seen in other subsurface environments (Anantharaman et al., 2016), Soudan MAGs contained portions of the pathway and likely specialize on portions of the pathway and rely on interspecies exchanges. Nitrogen fixation is a keystone process in the subsurface, as N_2_ must be recycled back to ammonia (Swanner and Templeton, 2011). We identified six MAGs containing the NifDHK suggesting the potential of fixing N_2_ to ammonia (Figure 6 and Supplementary Figure 3). However, it is unclear whether the *Methanolobus* MAGs are capable of diazotrophic growth is possible, as it is not universal for methanogens (Raymond et al., 2004). Nonetheless, there are several potential routes for nitrogen to be reintroduced. Finally, Soudan-2 is the only MAG predicted to couple nitrate reduction to thiosulfate oxidation (Supplementary Table 2).

### Iron cycling in Soudan Brines

Iron is a prominent feature of Soudan, which is prevalent within both the brine waters and in the surrounding minerals. The abundant iron in Soudan can act as a key electron acceptor or donor for iron reducing and iron oxidizing organisms, respectively, within the boreholes. Previous enrichments from Soudan have isolated microorganisms capable of iron oxidation, *Marinobacter subterrani* (Bonis and Gralnick, 2015), and iron reduction, *Desulfuromonas soudanensis* WTL (Badalamenti et al., 2016). To identify genes that may be involved in iron oxidation or reduction encoded in the Soudan MAGs, the bioinformatics tool FeGenie was used (Garber et al., 2020). FeGenie uses a combined Hidden Markov Model (HMM) and BLAST approach to look for known iron-related genes, including those associated with iron reduction and oxidation pathways. None of the MAGs contained genes reported to be associated with iron oxidation. This was expected considering the lack of oxygen or light within the borehole waters that would be needed for potential aerobic iron oxidation or photosynthetic iron oxidation pathways that FeGenie can predict (Garber et al., 2020). It should be noted that for iron cycling there is a large knowledge gap for identifying organisms capable of reducing or oxidizing Fe. For instance, *M. subterrani* has been shown previously to mediate iron oxidation (Bonis and Gralnick, 2015). However, this organism lacks Fe oxidizing genes. This disparity highlights there is likely unseen novelty in element cycling and suggest that Soudan-18, despite not containing iron cycling genes, may still contribute to this important cycle.

FeGenie predicted genes encoding putative iron reduction pathways in Soudan-6^∗^, Soudan-16 and Soudan-19. Sequence alignment of the bins of Soudan-6^∗^ to a well characterized strain of *Geobacter sulfurreducens* studied in the same lab that the metagenomic DNA was extracted in showed a sequence similarity of over 99% per bin tested. The high sequence similarity to a lab strain as well as the low completeness of Soudan-6^∗^ (41.56%, see Table 6) likely indicates that Soudan-6^∗^ was introduced during DNA processing and did not originate from the borehole waters. Additionally, the lack of the well-characterized *G. sulfurreducens* strain found in Soudan enrichments using similar growth strategies as well as its poor salt tolerance and fast growth rate relative to other Soudan isolates give further evidence that this bin is likely a contaminant. Although Soudan-6^∗^ is unlikely to have come from Soudan, FeGenie was still performed on the Soudan-6^∗^ bins to act as a positive control and validation of the bioinformatic tool. Soudan-6^∗^ was indeed predicted to have homologs of *omaB*, *omcC*, *omcF*, *omcS*, and *omcZ*, which have all been shown to be associated with iron reduction in *G. sulfurreducens* (Kim et al., 2005; Mehta et al., 2005; Nevin et al., 2009; Liu et al., 2014). The identification of these genes in the MAG gives highlights the utility of the FeGenie to identify genes associated with the reduction of ferric iron sources, such as Fe(III) oxides, Fe(III)-citrate, and ferrihydrite, as well as Mn(IV) oxides (Mehta et al., 2005; Inoue et al., 2010). Even if Soudan-6^∗^ did not originate from the Soudan borehole waters, the identification of genes associated with iron reduction in an incomplete MAG gives some validation of the use of FeGenie as a bioinformatics tool to study iron associated genes.

Soudan-16 carries homologs of both *mtrA* and *mtrB*, which are associated with iron reduction in *Shewanella* species as a part of the *mtrCAB* complex (Beliaev and Saffarini, 1998; Myers and Myers, 2002; Pitts et al., 2003). The MtrCAB protein complex facilitates electron transfer across the outer membrane (Coursolle et al., 2010), which enables reduction of extracellular substrates including soluble and insoluble forms of Fe(III). In this complex, the electron passes from a periplasmic electron carrier to the decaheme cytochrome MtrA, which is embedded within the transmembrane beta-barrel MtrB (Edwards et al., 2020). The electron is then transferred to the extracellular decaheme cytochrome MtrC before being donated to an extracellular acceptor (Hartshorne et al., 2007; Edwards et al., 2018). The lack of a predicted *mtrC* in the complete Soudan-16 MAG suggests that it is unable to reduce insoluble iron minerals, as *Shewanella oneidensis mtrC* knockout mutants were shown unable to reduce insoluble Fe(III) oxides (Coursolle et al., 2010). However, a *S. oneidensis* mutant lacking *mtrC* and 3 other extracellular cytochromes partly recovered reduction activity of chelated iron and manganese oxide through single point mutations in both *mtrA* and *mtrB* (Bücking et al., 2012), suggesting that *mtrC* may not be necessary to reduce iron in some backgrounds. A BLASTP search of the Soudan-16 predicted MtrA shows homology to DmsE, which is the decaheme periplasmic component of the dimethyl sulfoxide (DMSO) reductase complex in *S. oneidensis* (Gralnick et al., 2006) and has been shown to partly functionally replace MtrA during iron reduction (Coursolle and Gralnick, 2010). This homology is unsurprising as differentiating between the periplasmic decaheme cytochromes involved in iron reduction (MtrA and MtrD), DMSO reduction (DmsE), and proposed iron oxidation (PioA and MtoA) pathways is extremely difficult (Bewley et al., 2012). To determine the actual role of the predicted *mtrA* and *mtrB* genes in iron reduction, isolation and characterization of Soudan-16 is required.

Soudan-19 has homologs to *fmnA, fmnB, pplA, eetA, and eetB*, which are associated with the recently described iron reduction pathway in the Gram-positive bacteria *Listeria monocytogenes* (Light et al., 2018) and *Enterococcus faecalis* (Hederstedt et al., 2020). These pathways use a flavin mononucleotide (FMN) transferase, FmnB, to covalently attach FMN to an extracellular lipoprotein PplA, which is the terminal iron reductase. Interestingly, *L. monocytogenes* is a flavin auxotroph, which means that the FMN used for this pathway must be acquired from the environment (Light et al., 2018). Likewise, Soudan-19 is predicted to lack genes needed for flavin biosynthesis, indicating that Soudan-19 must acquire flavins from other organisms in the ecosystem. The high abundance (based on genome coverage) and even distribution of Soudan-19 throughout the three boreholes suggests that Soudan-19 could be an important and active organism within the Soudan boreholes.

### Hydrogen Cycling in Soudan Brines

Hydrogen is a versatile electron donor and acceptor and is a common currency in all environments [see references within (Greening and Boyd, 2020)]. In the subsurface, there are several (a)biotic processes that can generate and consume hydrogen (Sherwood Lollar et al., 2014; Gregory et al., 2019). Within subsurface lithoautotrophic microbial ecosystems, H_2_ consumption can maintain and drive microbial metabolisms (Lin et al., 2006; Lau et al., 2016) as well as act as a proxy for understanding dominant terminal electron accepting processes in subsurface aquifers (Lovley and Goodwin, 1988). Soudan brines, much like SURF (Osburn et al., 2014; Momper et al., 2017), are very low in H_2_ concentrations. At SURF, despite low concentrations of H_2_ many of the MAGs contained hydrogenases (Momper et al., 2017), suggesting hydrogen may be produced and consumed. At Soudan, we detected several MAGs with the capability of producing or consuming H_2_ (Supplementary Figure 3). This would suggest that like SURF, hydrogen consumption may result in extremely low concentrations in brines. Furthermore, microbial iron reduction is capable of drawing H_2_ concentrations below nanomolar concentrations (Lovley and Goodwin, 1988). Thus, at Soudan, where oxidized iron sources are not limiting, hydrogen may be rapidly consumed biotically or abiotically, which has overarching consequences for the structure of the microbial community and could constrain the metabolic activity of methanogens, i.e., hydrogenotrophic, acetoclastic, or methylotrophic.

### Biogeochemical Cycling Differs Between Boreholes

**FIGURE 7:**
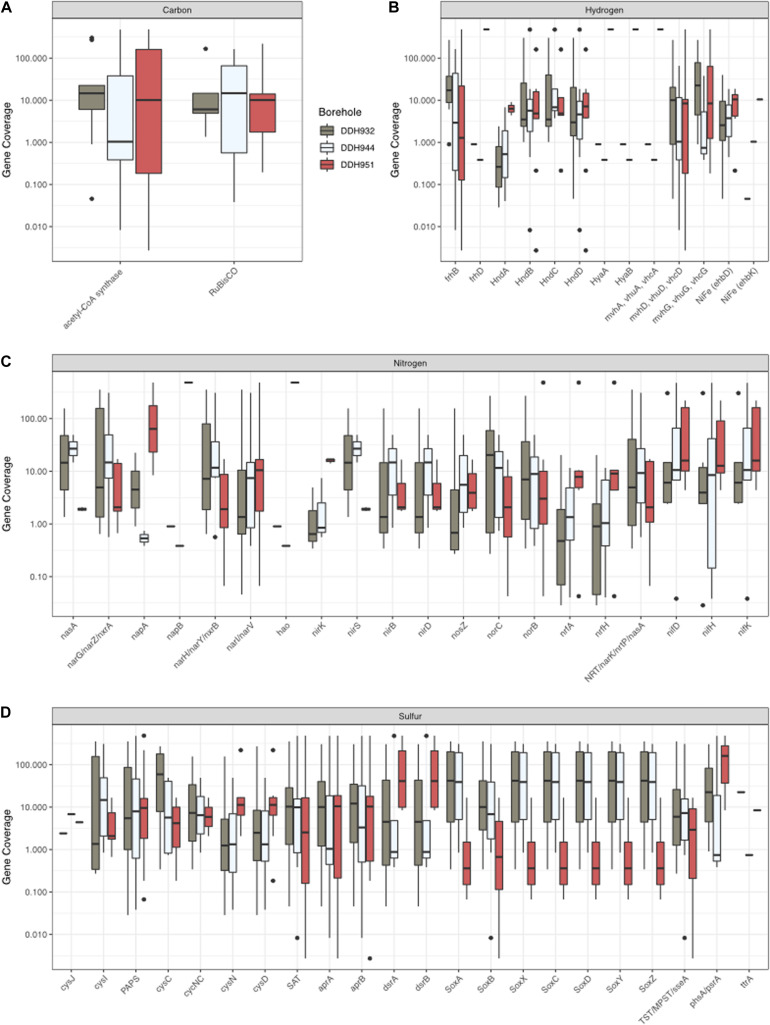
The dominance of genes for carbon **(A)**, hydrogen **(B)**, nitrogen **(C)**, and sulfur **(D)** at each borehole. Gene coverage is based on the MAG coverage.

In our system, there are subtle geochemical differences between boreholes DDH932, DDH944, and DDH951 (Table 1). However, these subtle differences have important impacts to the microbial community structure (Figures 2, 3) and the minerals that form within the boreholes and as the fluids exit (see Figure 1 for the different iron minerals that form at the borehole). As we’ve seen looking at the MAGs individually, there are sometimes large differences in coverage between the boreholes for a single MAG (Figure 3). These differences in abundances likely have larger implications for biogeochemical cycling when viewed from the perspective of the entire microbial community. Using the MAG coverage as a proxy for abundance of the gene, we can see that there are also notable differences in the nitrogen, sulfur, hydrogen and carbon cycles, such that boreholes DDH951 and DDH944 represent ends of a spectrum and DDH932 is a blend of both, as the presence of nearly all genes were detected and abundant in DDH932 (Figures 7A–D). For the nitrogen cycle, we see the *napA, nirK*, and *nrfA* occur primarily in DDH951, while other genes like *nifH* are more balanced amongst the boreholes (Figure 7C). Sulfur oxidation via the SOX system is primarily in DDH944 and DDH932, while sulfur reduction is primarily in DDH951 and DDH932 (Figure 7D). Similar trends with hydrogen are also seen, where hydrogenase diversity is greater in DDH951 and DDH932. Finally, the Wood-Ljungdahl pathway is only present in DDH951 and DDH932, suggesting that the isotopic fractionation of carbon in these boreholes may be different than DDH944, depending on the fixation rates of these pathways. Together, these differences have larger implications to the patchiness of the energetic landscape and ultimately biomass in the subsurface. Furthermore, enzyme cofactor availability may be an additional reason why we see differentially abundant gene systems, for example *nirK* nitrite reductase requires copper while *nirS* uses iron. While most cofactors are needed at low concentration in the cell, the rate of delivery of bioactive forms of necessary cofactors to the microbial community may be slow and thus may regulate the presence, absence and abundance of a microorganism.

### Conclusion

As has been seen in other subsurface systems (Chivian et al., 2008; Momper et al., 2017), individual microorganisms carry genes that allow them to be metabolically versatile and potentially couple several biogeochemical cycles. At Soudan we see multiple MAGs containing genes that could contribute to several element cycles, like C, N, S, and H. However, understanding whether these microorganisms are coupling these cycles together requires further study. The novelty of many of these genomes is interesting and suggests that other subsurface systems may harbor phylogenetically deep branching microorganisms. Further the role of viruses in this system has yet to be identified. Badalamenti et al. (2016) identified a prophage in their genome and Daly et al. (2019) show that viruses are highly active in fractured shales. We fully anticipate that viral lysis is an important process for releasing carbon back into the system at Soudan including glycine-betaine. The presence of only methyl-utilizing methanogens at Soudan is interesting and presents a unique opportunity to understand how microorganisms fractionate GB via fermentation reactions to produce TMA, which is ultimately converted to methane with an isotopic fractionation similar to abiotic production. The co-occurrence of GB cycling and methanogenesis in deep subsurface systems, is intriguing and suggests that these processes may be ubiquitous in deep saline brines. Finally, to understand the pervasiveness of these process in the deep subsurface, more of metagenome-based studies need to be performed in diverse terrestrial systems.

## Data Availability Statement

The datasets presented in this study can be found in online repositories. The names of the repository/repositories and accession number(s) can be found in the article/Supplementary Material.

## Author Contributions

CS processed DNA based data and wrote manuscript. JB collected physical samples and extracted DNA. JT sampled and analyzed gases, performed incubations, and contributed to the writing. DH, DB, and JG contributed to the writing and analyzed the MAGs for iron cycling. SA collected the long-term chemistry of brine fluids and contributed to the manuscript editing. BT contributed to the sampling and manuscript editing. All authors contributed to the article and approved the submitted version.

## Conflict of Interest

The authors declare that the research was conducted in the absence of any commercial or financial relationships that could be construed as a potential conflict of interest.

## Footnotes

1 https://vamps2.mbl.edu/portals/CODL

2 https://www.bioinformatics.babraham.ac.uk/projects/fastqc/

3 https://vamps2.mbl.edu
