# Supplementary material for: Novel Microbial Groups Drive Productivity in an Archean Iron Formation
Source: Front Microbiol. 2021 Mar 30;12:627595. doi: 10.3389/fmicb.2021.627595 (PMC8042283; doi:10.3389/fmicb.2021.627595)
Supplement: Supplementary file 1 [file Data_Sheet_1.pdf]

Supplementary Information

Novel microbial groups drive productivity in an Archean Iron formation.

Cody S. Sheik[1*], Jon Badalamenti[2,3], Jon Telling[4], David Hsu[3,5], Scott C. Alexander[6], Daniel Bond[3,5], Jeffrey A. Gralnick[3,5], Barbara Sherwood Lollar[7], Brandy M. Toner[6,8].

[1]Department of Biology and the Large Lakes Observatory, University of Minnesota Duluth, Duluth, MN, USA

[2]Univeristy of Minnesota Genomics Center, University of Minnesota – Twin Cities, Minneapolis Minnesota

[3]BioTechnology Institute, University of Minnesota - Twin Cities, St. Paul, MN, USA

[4]School of Natural and Environmental Sciences, Newcastle University, Newcastle-upon-Tyne, UK

[5]Plant and Microbial Biology, University of Minnesota - Twin Cities, St. Paul, MN, USA

[6]Department of Earth and Environmental Sciences, University of Minnesota – Twin Cities, Minneapolis, MN, USA

[7]Department of Earth Sciences, University of Toronto, Toronto, Ontario, Canada M5S 3B1

[8]Department of Soil, Water, and Climate, University of Minnesota - Twin Cities, St. Paul, MN, USA

*Corresponding Author
Cody S. Sheik
cssheik@d.umn.edu

SI Table 1. Detection limits for water element analysis.

Parameter	Method	Reference	Limit of Detection	Limit of Quantification	Accuracy
Temperature	Non-Hg thermometer	ASTM 63C			±0.1°C
pH	Ag/AgCl electrode	EPA 150.1			±0.02 unit
Conductivity		SM 2510	10□mhos	10mhos	±5%
Redox	Pt electrode				±50 mV
Diss. Oxygen	Optical DO probe		0.01 ppm	0.05 ppm	±2%
		**Cations**			
NH_4	IC	D6919-03	0.005 ppm	0.02 ppm	±2%
Al	ICP/OES	EPA 200.7	0.001 ppm	0.005 ppm	±2%
Ba	ICP/OES	EPA 200.7	0.001 ppm	0.005 ppm	±2%
Ca	ICP/OES	EPA 200.7	0.15 ppm	0.5 ppm	±2%
Fe	ICP/OES	EPA 200.7	0.03 ppm	0.10 ppm	±2%
K	ICP/OES	EPA 200.7	0.10 ppm	0.3 ppm	±2%
Mg	ICP/OES	EPA 200.7	0.05 ppm	0.2 ppm	±2%
Mn	ICP/OES	EPA 200.7	0.003 ppm	0.010 ppm	±2%
Na	ICP/OES	EPA 200.7	0.05 ppm	0.2 ppm	±2%
P	ICP/OES	EPA 200.7	0.010 ppm	0.3 ppm	±2%
Si	ICP/OES	EPA 200.7	0.10 ppm	0.3 ppm	±2%
Sr	ICP/OES	EPA 200.7	0.001 ppm	0.005 ppm	±2%
		**Anions**			
Br	IC	EPA 300.0	0.002 ppm	0.006 ppm	±2%
Cl	IC	EPA 300.0	0.05 ppm	0.05 ppm	±2%
F	IC	EPA 300.0	0.002 ppm	0.006 ppm	±2%
NO_2-N	IC	EPA 300.0	0.001 ppm	0.005 ppm	±2%
NO_3-N	IC	EPA 300.0	0.001 ppm	0.05 ppm	±2%
P	ICP/OES	EPA 200.7	0.010 ppm	0.3 ppm	±2%
PO4-P	IC	EPA 300.0	0.005 ppm	0.015 ppm	±2%
S_2O_3	IC	EPA 300.0	0.002 ppm	0.010 ppm	±2%
SO_4	IC	EPA 300.0	0.05 ppm	0.15 ppm	±2%

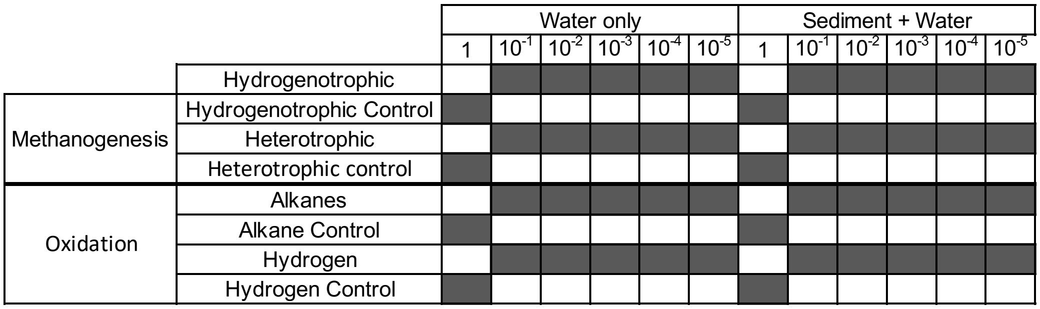

SI Figure 1. Design of the MPN experiments, each box represents triplicate bottles.

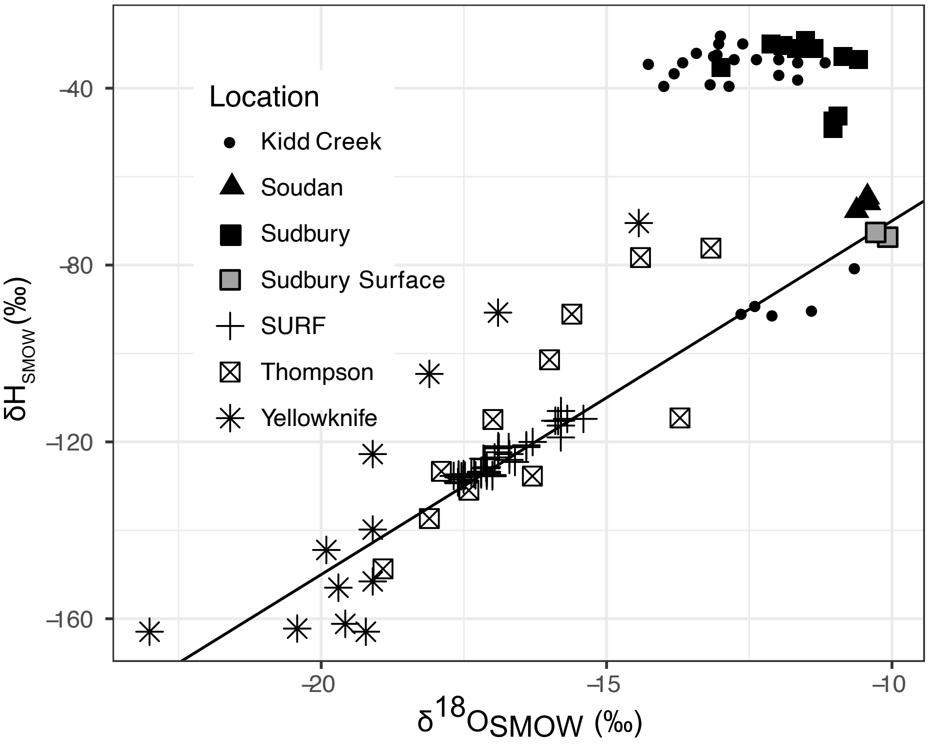

SI Figure 2. Comparison of oxygen and hydrogen isotopes of water from North American mines. For most mine sites, the symbols falling on the line represent mixing with surface waters (Li et al., 2016; Sherwood Lollar et al., 2019).

SI Table 2. Phylogenetic placement and quality of Metagenome Assembled Genomes (MAGs) recovered from metagenomic assembles. Table is sorted by completeness.

				GTDB-TK Phylogenetic Classification						
**Genome**	**Completeness**	**Contamination**	**Strain Heterogeneity**	**Domain**	**Phylum**	**Class**	**Order**	**Family**	**Genus**	**Species**
Soudan-13_2	98.53	0.5	50	Bacteria	Actinobacteriota	Actinobacteria	Actinomycetales	Demequinaceae	*Demequina*	Novel
Soudan-13_1	96.92	0	0	Bacteria	Actinobacteriota	Actinobacteria	Actinomycetales	Demequinaceae	*Demequina*	Novel
Soudan-30	95.26	2.01	0	Bacteria	Actinobacteriota	Thermoleophilia	RBG-16-64-13	RBG-16-64-13	Novel	Novel
Soudan-9	98.82	2.72	0	Bacteria	Bacteroidota	Bacteroidia	Bacteroidales	Marinilabiliaceae	*Marinilabilia*	Novel
Soudan-14	43.12	1	66.67	Bacteria	Bacteroidota	Bacteroidia	Flavobacteriales	Weeksellaceae	*Cloacibacterium*	normanense
Soudan-16	100	1.79	0	Bacteria	Desulfobacterota	Desulfobulbia	Desulfobulbales	Desulfocapsaceae	Novel	Novel
Soudan-6	41.56	0.65	0	Bacteria	Desulfuromonadota	Desulfuromonadia	Geobacterales	Geobacteraceae	*Geobacter*	sulfurreducens
Soudan-7	40.87	0	0	Bacteria	Desulfuromonadota	Desulfuromonadia	Desulfuromonadales	Pelobacteraceae_A	Novel	Novel
Soudan-22	99.29	0.35	0	Bacteria	Firmicutes_A	Clostridia	Peptostreptococcales	Novel	Novel	Novel
Soudan-15	97.67	12.11	0	Bacteria	Firmicutes_A	Clostridia	Tissierellales	Dethiosulfatibacteraceae	UBA8670	Novel
Soudan-1	90.09	0.38	25	Bacteria	Firmicutes_B	Desulfitobacteriia	Desulfitobacteriales	Desulfitobacteriaceae	*Desulfosporosinus*	Novel
Soudan-25	20.44	0.05	0	Bacteria	Firmicutes_B	Desulfitobacteriia	Desulfitobacteriales	Syntrophobotulaceae	*Gracilibacter*	Novel
Soudan-19	99.27	2.04	0	Bacteria	Firmicutes_B	Desulfotomaculia	Desulfotomaculales	Novel	Novel	Novel
Soudan-17	98.92	3.57	0	Bacteria	Firmicutes_B	Moorellia	Novel	Novel	Novel	Novel
Soudan-20	98.41	1.29	25	Bacteria	Firmicutes_B	Moorellia	UBA1874	Novel	Novel	Novel
Soudan-29	98.68	0	0	Bacteria	Firmicutes_F	Halanaerobiia	Halanaerobiales	Halanaerobiaceae	Novel	Novel
Soudan-4	85.57	2.05	25	Bacteria	Firmicutes_F	Halanaerobiia	Halanaerobiales	Novel	Novel	Novel
Soudan-3	73.75	1.83	75	Bacteria	Firmicutes_F	Halanaerobiia	Halanaerobiales	DTU029	Novel	Novel
Soudan-8	62.83	2.85	60	Bacteria	Firmicutes_F	Halanaerobiia	Halanaerobiales	DTU029	Novel	Novel
Soudan-5	46.55	1.72	0	Bacteria	Firmicutes_F	Halanaerobiia	Halanaerobiales	DTU029	Novel	Novel
Soudan-27	100	1.96	0	Archaea	Halobacterota	Methanosarcinia	Methanosarcinales	Methanosarcinaceae	*Methanolobus*	Novel
Soudan-28	99.84	0.65	0	Archaea	Halobacterota	Methanosarcinia	Methanosarcinales	Methanosarcinaceae	*Methanolobus*	Novel
Soudan-21	71.68	7.92	14.29	Bacteria	Patescibacteria	Paceibacteria	UBA6257	UBA6257	Novel	Novel
Soudan-12_2	97.27	0	0	Bacteria	Proteobacteria	Alphaproteobacteria	Rhodobacterales	Rhodobacteraceae	*Confluentimicrobium*	Novel
Soudan-10	92.78	1.47	50	Bacteria	Proteobacteria	Alphaproteobacteria	Rhodobacterales	Rhodobacteraceae	*Roseovarius*	Novel
Soudan-11	78.97	1.72	100	Bacteria	Proteobacteria	Alphaproteobacteria	Rhodobacterales	Rhodobacteraceae	*Confluentimicrobium*	Novel
Soudan-12_1	100	0	0	Bacteria	Proteobacteria	Gammaproteobacteria	Halothiobacillales	Halothiobacillaceae	WRN-7	Novel
Soudan-18	98.38	0.88	66.67	Bacteria	Proteobacteria	Gammaproteobacteria	Pseudomonadales	Oleiphilaceae	*Marinobacter*	*subterrani*
Soudan-2	36.99	0	0	Bacteria	Proteobacteria	Gammaproteobacteria	Pseudomonadales	Oleiphilaceae	*Marinobacter*	*guineae*

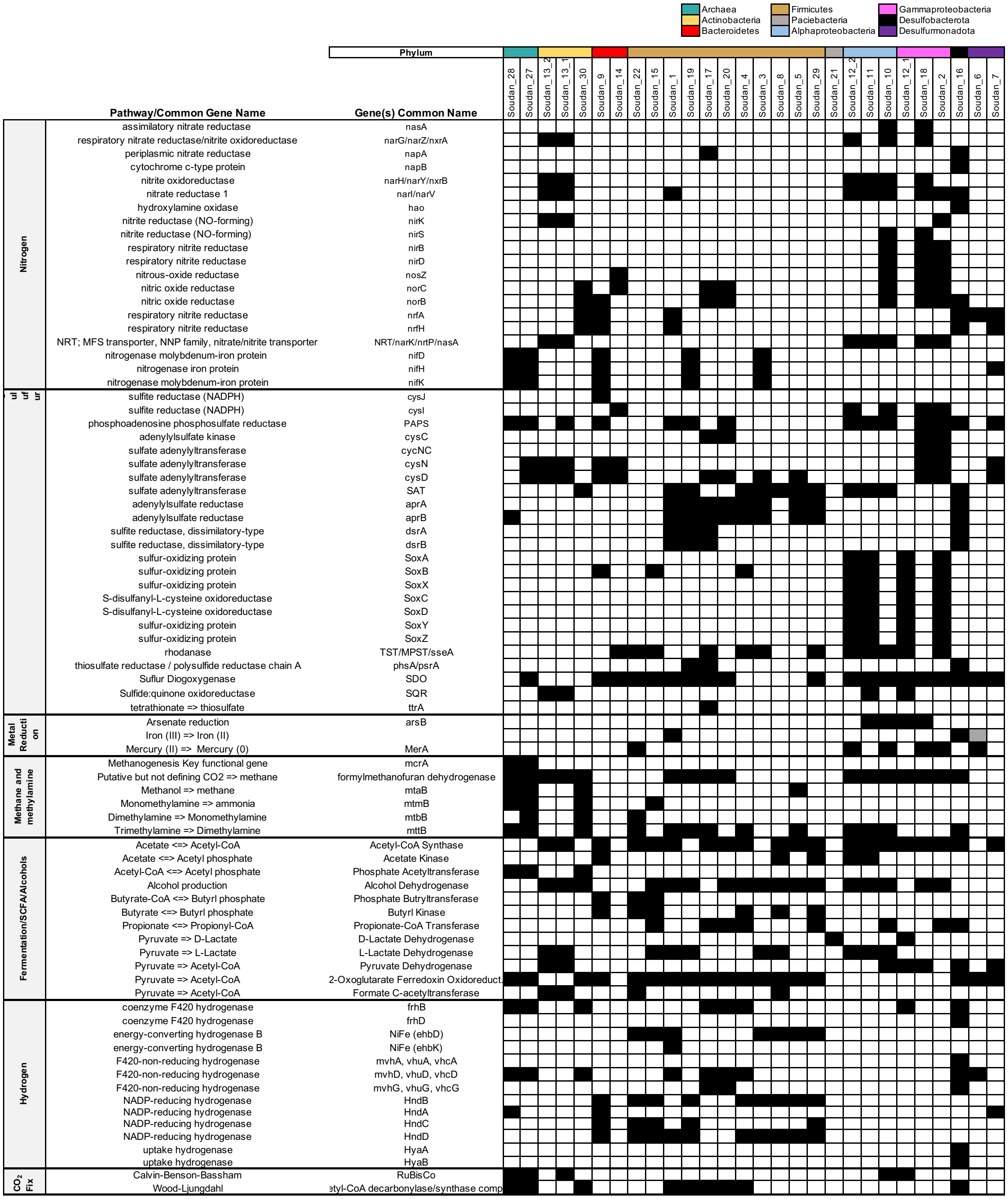

SI Figure 3. Metabolism overview of recovered MAGs. Filled in squares indicates the presence of the gene.
